# Isolation of coagulase-positive staphylococci from bitches’ colostrum and milk and genetic typing of methicillin-resistant *Staphylococcus pseudintermedius* strains

**DOI:** 10.1186/s12917-015-0490-x

**Published:** 2015-07-23

**Authors:** Ada Rota, Michela Corrò, Ilenia Drigo, Alessio Bortolami, Stefan Börjesson

**Affiliations:** Department of Veterinary Science, University of Turin, Largo Paolo Braccini 2, 10090 Grugliasco, TO Italy; Istituto Zooprofilattico Sperimentale delle Venezie, viale dell’Università 10, 35020 Legnaro, PD Italy; Department of Animal Health and Antimicrobial Strategies, National Veterinary Institute (SVA), SE-751 89 Uppsala, Sweden

**Keywords:** Coagulase-positive staphylococci, MRSP, Dog, Colostrum, Milk

## Abstract

**Background:**

Among the coagulase-positive, potentially pathogenic staphylococci, *Staphylococcus pseudintermedius* has been frequently isolated from bitches’ milk. This organism colonizes the mammary gland or causes infection, while *S. aureus* has been only occasionally reported. The objective of this study was to investigate the occurrence and persistence of coagulase-positive staphylococci in the colostrum and milk of postpartum bitches, either treated or untreated with antimicrobials, and to assess the incidence, antibiotic resistance profile and genetic type of the methicillin-resistant strains.

On postpartum D1, D7 and D15, drops of secretion were collected from the mammary glands of 27 postpartum bitches, nine of which were treated with antimicrobials. Coagulase-positive staphylococci were identified, antimicrobial susceptibility and the presence of *mec*A were tested and the genetic profile of methicillin-resistant strains was assessed.

**Results:**

*Staphylococcus pseudintermedius* was the only coagulase-positive staphylococcus isolated, and its presence was detected in 21 out of 27 bitches and in 66 out of 145 swabs. In a single bitch, it caused puerperal mastitis. In untreated bitches, the frequency of isolation was lower in colostrum than in milk. All of the isolates except one were resistant to at least three antimicrobial classes, while 14 out of 66 *S. pseudintermedius* strains were methicillin-resistant *mec*A positive (MRSP) and were isolated from eight bitches housed in the same breeding kennel. A significant association was found between antimicrobial treatment and the presence of MRSP. Six of the 12 typed isolates belonged to *spa*-type t02 carrying SCCmec II/III, and another six were non-typeable with *spa* carrying SCCmec IV. The t02-SCCmec II/III isolates were sequence type (ST) 71; four NT-SCCmec IV isolates were ST258 and two were ST369. PFGE showed that isolates from the same dog had identical band patterns, while isolates from different dogs had unique band patterns. MRSP strains showed multidrug resistance profiles.

**Conclusions:**

Our results confirm that *S. pseudintermedius* is the most frequently isolated coagulase-positive staphylococcus from bitches’ milk. The isolation of several different strains of MRSP with different genetic characteristics in the same kennel and the fact that two of the strains belonged to a sequence type (ST) described for the first time are noteworthy findings.

## Background

Staphyloccocci are commonly isolated from milk samples of healthy bitches, colonizing the mammary gland without apparently affecting puppy mortality [1]. However, they are also known agents of acute and subclinical mastitis [2, 3]. Among the coagulase-positive potentially pathogenic staphylococci, *Staphylococcus pseudintermedius* has been frequently isolated from bitches’ milk [1, 4], while *S. aureus* has been only occasionally reported [3].

Dogs are considered natural hosts of *S. pseudintermedius*, which is one of the common opportunistic pathogens of the species, frequently associated with skin, ear and wound infections [5, 6]. Methicillin-resistant *S. pseudintermedius* (MRSP) strains have been isolated with increasing frequency both from healthy and diseased dogs in the past few years. Moreover, these strains often show resistance towards many other antimicrobial agents [7–9]. In veterinary settings, MRSP is an important nosocomial pathogen and represents a veterinary hazard because of the few therapeutic options available for treatment [10, 11]. Moreover, antimicrobial use is a risk factor for MRSP colonization and infection of dogs [12–14]. In addition, a strong association has been found between previous hospitalization and MRSP colonization [12] or infection [15]. Although the population structure of *S. pseudintermedius* appears to be extremely heterogeneous [6], current data showed that clone ST71 is dominant in Europe among the methicillin-resistant strains [16]. However, studies about MRSP carriage patterns and persistence are very scarce [14].

This study aimed to investigate the occurrence and persistence of coagulase-positive staphylococci in the colostrum and milk of postpartum bitches, either treated or untreated with antimicrobials, focusing on methicillin-resistant strains.

## Methods

### Ethics statement

All biological material used to perform the present study was collected for diagnostic purposes. The study was performed in accordance with the guidelines for the care and use of animals of the Department of Veterinary Science of the University of Turin. Previous informed consent was obtained by the owners.

### Animals and sampling

The study was carried out from July 2012 to July 2013 and included samples collected from 27 postpartum bitches of different breeds, either housed in two breeding kennels (Kennel 1: *N* = 12; Kennel 2: *N* = 8) or privately owned (*N* = 7). Twenty bitches whelped spontaneously, while seven underwent Caesarean section and were prophylactically treated with cephazolin during the following 5 days. On postpartum day 1, 7 and 15, samples of colostrum (D1) and milk (D7 and D15) were collected from the inguinal mammary glands for cytological and bacteriological exams, in order to assess health status and to diagnose overt or subclinical mastitis. For bacteriological samples, after cleaning the area with saline solution and drying it with a clean towel, each gland was milked; the first drops of secretion were discarded, and the following 3–4 drops were intercepted on a bacteriological swab and placed in Amies medium (Copan Innovation, Brescia, Italy).

The health conditions of the mammary glands and pups were checked daily, and neonatal mortality was recorded at D15.

### Isolation and identification of coagulase-positive staphylococci

Swabs were seeded on Blood Agar Base N°2 (Biolife, Milano, Italy) with 5 % defibrinated sheep blood (Allevamento Blood, Teramo, Italy) and inoculated in broth containing 6.5 % sodium chloride (Mueller-Hinton broth, Biokar Diagnostics, Alonne, FR). After an incubation period at 37 °C ± 1 °C for 18–24 h, up to five colonies were recovered from blood agar for preliminary identification as coagulase positive staphylococci. Preliminary identification was based on colony morphology, gram stain appearance, catalase test, hemolysis, pigment production and coagulase enzymatic activity, evaluated by a tube coagulase test with rabbit plasma (Istituto Zooprofilattico delle Venezie, Legnaro, Italy).

The pre-enrichment broth was seeded in a methicillin-resistant staphylococci selective medium, CHROMagar® MRSA II (BD BBL™, New Jersey, USA). Selective plates were incubated at 35 °C ± 1 °C for 24–48 h in aerobic conditions. Growth on CHROMagar® MRSA II as pale pink colonies represented the first sign of methicillin resistance. Coagulase positivity was also tested.

Coagulase positive staphylococci species identification was performed by a MALDI-TOF MS: Microflex LT instrument (MALDI Biotyper, Bruker Daltonics) equipped with FlexControl software (version 3.3, Bruker Daltonics).

### Antimicrobial susceptibility testing

Colonies grown on blood agar and on CHROMagar® MRSA II were tested for susceptibility to sixteen antimicrobial agents. The test was performed by the disk diffusion method on Mueller-Hinton agar (KIMA S.A.S, Padova, Italy) according to the guidelines of the Clinical Laboratory Standards Institute (CLSI) [17, 18]. Oxacillin was used as a marker to detect mecA-mediated methicillin resistance [17, 19].

Discs of penicillin G (10 IU), ampicillin (10 μg), amoxicillin-clavulanic acid (20 + 10 μg), oxacillin (1 μg), cefalotin (30 μg), spiramycin (100 μg), erythromycin (15 μg), tetracycline (30 μg), tilmicosin (15 μg), tylosin (30 μg), enrofloxacin (5 μg), licosamides (clindamycin CC 2 μg), tiamulin (30 μg), trimethoprim-sulfamethoxazole (1.25 + 23.75 μg) (BD BBL, New Jersey, USA), and cefquinone (30 μg) (Oxoid Ltd, Basingstoke, UK) were used for the antimicrobial sensitivity test. Interpretive criteria for the inhibition zone diameters provided by CLSI [17, 18], or alternatively by the manufacturers (i.e., tylosin), were followed.

Isolates resistant to three or more classes of antimicrobial agents were considered multidrug-resistant [20].

### Identification of MRSP

Oxacillin-resistant *S. pseudintermedius* strains were confirmed as MRSP after the detection of the *mec*A gene by PCR [19]. *Staphylococcus aureus* DSMZ 11729 was used as a control.

### *Genetic typing of methicillin-resistant S. pseudintermedius (MRSP) strains [Pulsed-field gel electrophoresis* (*PFGE), spa-typing,* SCC*mec typing and Multilocus Sequence Typing (MLST)]*

*spa*- typing was performed according to the scheme developed by Moodley et al. [21] using sequence signatures 5’-AATAATTCA and 3’-GACAAGCG [8]. Multilocus Sequence Typing (MLST) was performed according to Solyman et al. [22].

SCC*mec* types I–V, SCC*mec* III lacking SCC-Hg, II-III, and VII-241 were determined as previously described [23].

Preparation of chromosomal DNA and plugs (Agarose Prep; Amersham Biosciences, Uppsala, Sweden) for pulsed-field gel electrophoresis (PFGE) were performed according to the Harmony protocol [24], and *S. aureus* NCTC8325 was used as a control. DNA was fragmented using 20U SmaI (Fermentas, Vilnius, Lithuania), and the fragments were separated in a CHEF-DR-II system (BIO-Rad Lab, Hercules, CA) with a 1.2 % agarose gel (Agarose NA; GE Healthcare, Uppsala, Sweden). The gel was run for 24 h at 5.6 V cm^-1^ with pulsed-time ramping 2–5 s at 14 ° C. The analysis of the fragment pattern was performed in BioNumerics® version 7.1 (Applied Maths, Gent, Belgium) on fragments between 9 and 117 kb using the Dice coefficient and Unweighted Pair Group Method with Arithmetic Mean cluster analysis, with position optimization set at 0.5 % and tolerance at 1.2 %.

### Analysis of data

The frequency of isolation of coagulase-positive staphylococci and of methicillin-resistant strains was compared between bitches that had been treated or untreated with antimicrobials (Fisher’s exact test) and among successive samples (Chi-squared test). The same test was used to assess the association between antimicrobial treatment and isolation of methicillin-resistant strains. The analysis of data was performed with GraphPad Prism software (GraphPad Software 4.00, California, USA.). *P* < 0.05 was considered statistically significant.

## Results

A total of 145 swabs were collected from the 27 bitches and were cultured. One bitch showed acute mastitis at D1, and another bitch had a vulvar abscess and generalized dermatitis. Both of them were treated with cephazolin from D1 to D7, bringing the number of treated bitches to nine.

From the nine treated bitches, 16 samples of foremilk were collected at D1 (2 mammary glands of different bitches were empty), 17 of milk at D7 (a mammary gland was empty) and 14 of milk at D15 (2 bitches could not be sampled). From the 18 untreated bitches, 34 samples of foremilk were collected at D1 (2 mammary glands of different bitches were empty), 34 of milk at D7 (2 mammary glands of different bitches were empty) and 30 of milk at D15 (3 bitches could not be sampled).

All of the coagulase-positive staphylococci were identified as *S. pseudintermedius.* Coagulase-positive staphylococci were present in 45.5 % of samples, with 21 out of 27 bitches positive during at least one sampling time. Only six bitches were persistently positive; seven bitches were positive at two different sampling times and eight were positive only on one occasion. The pattern of isolation of *S. pseudintermedius* is reported in Table 1.

**Table 1:** Frequency of isolation of *S. pseudintermedius* at post-partum D1, D7 and D15 from bitches that received or did not receive antibiotic treatment in the first week post-partum

	D1	D7	D15
Treated bitches (*N* = 9)	2/16	7/17	7/18
Non treated bitches (*N* = 18)	11/34^(a)^	20/34^(b)^	19/30^(c)^

^(a), (b), (c)^indicate significant differences (*P* < 0.05)

The frequency of isolation of *S. pseudintermedius* was not significantly different between bitches that received antibiotics or not. Colostrum yielded a lower percentage of *S. pseudintermedius* isolates, but the difference among D1, D7 and D15 isolates was significant only for untreated bitches (*P* = 0.02).

Almost all of the isolates were multiresistant, and a single untreated bitch had *S. pseudintermedius* strains that were resistant to less than three antimicrobial classes. Fourteen *S. pseudintermedius* strains (21.2 %) were methicillin-resistant, carried the *mec*A gene and were isolated from eight different bitches, all belonging to Kennel 1, six of which were treated with antimicrobials. A significant association was found between antimicrobial treatment and the presence of MRSP (*P* = 0.0044).

The pattern of antimicrobial susceptibility of the 52 methicillin-susceptible *S. pseudintermedius* (MSSP) strains and of the 14 MRSP strains appears in Table 2.

**Table 2:** Antimicrobial Susceptibility Test (Kirby Bauer method): results of methicillin-susceptible *S. pseudintermedius* (MSSP) and methicillin-resistant *S. pseudintermedius* (MRSP) strains

Antimicrobials	S		I		R	
	MSSP (%)	MRSP (%)	MSSP (%)	MRSP (%)	MSSP (%)	MRSP (%)
Penicillin	0	0	0	0	100	100
Ampicillin	1.9	0	0	0	98.1	100
Amoxicillin-clavulanic acid	94.2	0	0	0	5.8	100
Oxacillin	100	0	0	0	0	100
Cefalotin	100	0	0	0	0	100
Cefquinone	96.2	0	3.8	0	0	100
Spiramycin	46.1	0	5.8	0	48.1	100
Tilmicosin	92.3	50	1.9	0	5.8	50
Tylosin	46.1	0	5.8	0	48.1	100
Enrofloxacin	100	35.7	0	7.1	0	57.1
Clindamycin	49.9	0	28.9	38.4	21.2	57.1
Trimethoprim-sulfamethoxazole	44.2	7.1	0	0	55.8	92.9
Tetracycline	42.3	7.1	0	0	57.7	92.9
Trimethoprim-sulfamethoxazole	67.3	35.7	0	0	32.7	64.3
Tiamulin	98.1	100	0	0	1.9	0

Sensitive (S), Intermediate (I), Resistant (R) were classified according to CLSI guidelines (CLSI 2013 a,b)

**Fig. 1:**
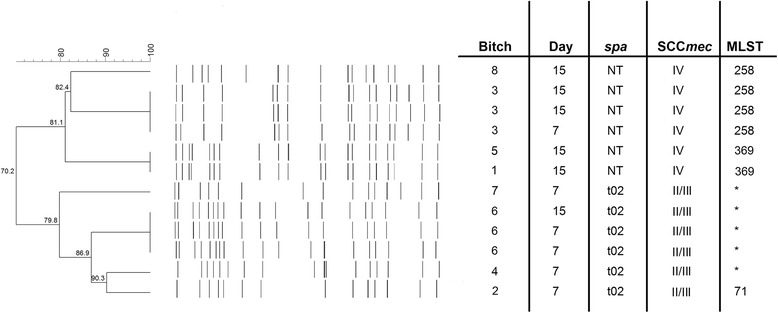
Typing of strains (*N* = 12). *denotes strains not subjected to MLST

Genetic typing was performed on 12 of the 14 MRSP isolates, and the typing results appear in Fig. 1. A single isolate was tested in bitch n.1 (Fig. 1) because the isolate at D1 could not be analyzed, and only one of the two isolates at D15 was submitted for analysis.

Out of the twelve MRSP isolates, six were *spa*-type t02 carrying SCCmec II/III and six were non-typeable (NT) with *spa* and carried SCCmec IV. One of the t02-SCCmec II/III isolates was subjected to MLST and was sequence type (ST) 71. All of the NT-SCCmec IV isolates were subjected to MLST; four isolates were ST258, while two isolates were ST369.

The six isolates belonging to t02-SCCmec II/III-ST71 were isolated from four different bitches, and three were from the same animal. The three isolates from the same bitch showed identical band patterns when using PFGE, whereas the other three t02-SCCmec II/III-ST71 showed unique band patterns. The four NT-SCCmec IV- ST258 strains were isolated from two bitches: three strains from one bitch, suffering from dermatitis and a vulvar abscess, had identical PFGE patterns, while the other NT-SCCmec IV- ST258, belonging to another bitch, differed from the other three in five bands and was isolated three months later. The two NT-SCCmec IV- ST369 strains, showing identical PFGE patterns, were isolated from two dogs, one of them suffering from mastitis. The time interval between the two isolations was two months. Table 3 shows the temporal sequence of isolation of the strains.

**Table 3:** Temporal sequence of isolation of the different genetic types of MRSP from colostrum (D1) and milk (D7, D15) of eight bitches housed in the same kennel and receiving or not receiving antibiotic treatment during 5–7 days postpartum (time interval July 2012-July 2013)

Bitch	Ab	D1		D7		D15	
		Right	Left	Right	Left	Right	Left
1)	NO	X				NT-SCCmec IV- ST369	X
4)	YES			t02-SCCmec II/III-ST71			
2)	NO			t02-SCCmec II/III-ST71			
5)	^a^YES						NT-SCCmec IV- ST369
6)	YES			t02-SCCmec II/III-ST71	t02-SCCmec II/III-ST71	t02-SCCmec II/III-ST71	
7)	YES			t02-SCCmec II/III-ST71			
3)	^b^YES			NT-SCCmec IV- ST258		NT-SCCmec IV- ST258	NT-SCCmec IV- ST258
8)	YES						NT-SCCmec IV- ST258

*Ab* antibiotic administration

^a^bitch showing mastitis

^b^bitch showing vulvar abscess and dermatitis

Both MSSP and MRSP were frequently isolated in one or more samples of healthy bitches with healthy and normally growing pups. Spontaneous deliveries (*N* = 20) yielded 101 pups, while 36 pups were born by Caesarean section. The overall mortality rate was 3 % among naturally born puppies and 11.1 % among Caesarean born puppies. All of the mortalities occurred within 72 h. The mortality rate in MSSP-positive bitches was 3.4 % in spontaneous deliveries (*N* = 58 pups). From the bitches that underwent Caesarean section, only MRSP was isolated, and the mortality rate was 8 % (5 bitches, 24 pups), while the mortality rate in MRSP-positive bitches whelping naturally was 3.2 % (*N* = 24 pups). The number of bitches is too low to compare values in the sub-groups.

## Discussion

*Staphylococcus pseudintermedius* was the only coagulase-positive staphylococcus isolated from bitches’ milk in our study, confirming previous investigations that reported this species as the most frequently isolated [1, 4]. In addition, our study examined day-one colostrum and showed that the frequency of *S. pseudintermedius* isolation was lower than in the following milk samples, at least in bitches that did not receive antibiotics. The colonization of mammary glands is likely influenced by feeding pups, and it is well-known that mammary gland infection by *S. pseudintermedius* is usually ascending [25]. The presence of *S. pseudintermedius* apparently did not affect mammary gland health or pup health, apart from one single case of puerperal mastitis. Our study is in agreement with Kuhn et al. [1], who reported similar findings. In the case of mastitis, previous observations showed that when only the mammary gland is involved, no detrimental effects are observed in pups [25].

It is rather alarming that almost all MSSP strains were multidrug-resistant [20], although they had high susceptibility rates (>90 %) towards cephalosporins, *β*-lactams potentiated with *β*-lactamase inhibitors and quinolones.

The high prevalence of MRSP isolates and of positive bitches in the current study was somewhat unexpected. However, MRSP occurrence may be strongly biased, being limited to eight animals sharing the same kennel environment with most receiving antibiotic treatment. The administration of antimicrobials to six out of the eight bitches is likely to have favored the selection of MRSP strains, although MRSP was also isolated from two untreated bitches at day 7 and at day 15. Aside from decreased susceptibility to *β*-lactamans, the MRSP isolates also showed resistance to macrolides, lincosamides, tetracyclines, and quinolones. The only antimicrobial that was always effective against MRSP was tiamulin. Perreten et al. [16] reported similar high percentages of resistance to tetracyclines, macrolides and lincosamides but a higher percentage of resistance to quinolones. Despite the antibiotic-resistance profile of the MRSP isolates, the mastitis, abscess and dermatitis resolved uneventfully, suggesting that laboratory susceptibility results may be different from field results and that the host defense systems may play a role in recovery.

The study revealed that several different MRSP strains were present in the same kennel. Furthermore, the same bitch carried the same strain at different times, while isolates from different animals showed unique PFGE patterns. Six of the isolates belong to the Europen major clonal lineage ST71-J-t02-II–III [16], but the other six isolates were non-typeable with *spa* and were shown to carry SCCmec IV*.* Four of these isolates belonged to the MLST ST258 (ST106 according to the old MLST 5-alleles scheme), which has previously been described as an emerging clone in Norway and Denmark [26, 27] and has also been described at a French veterinary clinic [28]. The remaining two isolates were shown to belong to a new MLST, designated ST369. Two bitches carried this new ST369- NT-IV MRSP, which were indistinguishable by PFGE. The first bitch carrying this strain was not usually living in the kennel but had been brought there for parturition. The other strain was isolated two months later from the milk of a bitch that underwent Caesarean section and suffered from acute mastitis. Direct contact between whelping and post-partum bitches was not allowed in the kennel, but all of them were housed in the nursery, although in individual boxes, so environmental or tool contamination cannot be excluded. The same observation can be made for the ST258- NT-IV isolates coming from two different bitches without direct contact which were swabbed at a three month interval.

There are few studies reporting data on carriage of MRSP in dogs; carriage for several months was detected in dogs previously treated for different conditions (post-operative infection, dermatitis, traumatic injuries, secondary infection), and it has been suggested that MRSP can establish itself as part of the normal bacterial flora, preventing total eradication [29].

From our study, we can conclude that *S. pseudintermedius* can be largely isolated from bitches’ mammary secretions without having a pathological significance on the health conditions of the mammary glands and nursing pups. Multidrug resistance frequently occurred in *S. pseudintermedius*, and the use of antimicrobials was associated with the occurrence of MRSP strains. More studies, involving greater numbers of animals, are needed in order to investigate the carriage and persistence of MRSP and also to focus on genetic traits of MRSP isolates. Notwithstanding the limits of this study, this research can help veterinarians and breeders maintain proper management of canine health in kennels.

## Conclusions

Our investigation provided valuable information regarding the occurrence, persistence and clinical importance of *S. pseudintermedius*, and also of MRSP strains, in bitches’ colostrum and milk.

In addition, the isolation of several strains of MRSP with different genetic characteristics in the same kennel is worth attention, as is the fact that two of the strains belonged to a sequence type (ST) identified for the first time.
